# Spontaneous Coronary Artery Dissection in a 49-Year-Old Woman Following Sexual Activity: A Case Report

**DOI:** 10.7759/cureus.108042

**Published:** 2026-04-30

**Authors:** John P Martinez Ponce, Oskar Ubysz, Anthony Costa, Lina Ataya, Sandeep K Bhangu, George Yassa, Frank Tilli

**Affiliations:** 1 Internal Medicine, Henry Ford Genesys Hospital, Grand Blanc, USA; 2 Cardiology, Henry Ford Genesys Hospital, Grand Blanc, USA; 3 Internal Medicine, Henry Ford Health, Grand Blanc, USA; 4 Cardiology, Henry Ford Health, Grand Blanc, USA; 5 Interventional Cardiology, Henry Ford Genesys Hospital, Grand Blanc, USA

**Keywords:** acute coronary syndrome, coronary artery intervention, exogenous hormones, multiparity, primary percutaneous coronary intervention (pci), spontaneous coronary artery dissection

## Abstract

Spontaneous coronary artery dissection (SCAD) is a rare but significant cause of acute coronary syndrome (ACS), particularly among middle-aged women. Unlike atherosclerotic ACS, SCAD is characterized by the separation of the coronary artery wall layers, leading to intramural hematoma formation and potential myocardial infarction. While SCAD is often associated with emotional or physical stressors, its pathophysiology remains incompletely understood, with hormonal, genetic, and arteriopathic factors playing significant roles. Here, we present a case of a 49-year-old woman with a history of hypertension, obesity, and prediabetes who developed SCAD following sexual activity. She presented with acute chest pain, ST-segment elevations, and reduced left ventricular ejection fraction (LVEF). Coronary angiography confirmed a type 2 SCAD involving the left anterior descending artery, left main, and left circumflex arteries. Conservative management with temporary mechanical circulatory support was pursued due to reduced LVEF. This case underscores the importance of recognizing SCAD in atypical presentations and highlights the role of conservative management strategies to optimize patient outcomes.

## Introduction

Spontaneous coronary artery dissection (SCAD) is a rare and underrecognized cause of acute coronary syndrome (ACS), presenting significant diagnostic and therapeutic challenges. SCAD primarily affects women, particularly those between 45 and 55 years of age, and has been linked to emotional or physical stressors such as intense exercise, labor, or sexual activity [1]. Unlike atherosclerotic ACS, which results from plaque rupture or thrombosis, SCAD is characterized by spontaneous separation of the coronary arterial wall layers, leading to intramural hematoma formation that may compromise luminal integrity and cause myocardial ischemia or infarction [1,2].

The underlying pathophysiology of SCAD is multifactorial and involves mechanical stressors, arteriopathies, and hormonal influences. SCAD has been reported to account for approximately 1%-3% of all ACS cases overall, but this proportion increases to 11%-13% among women presenting with ACS, particularly in younger and middle-aged populations [3]. Notably, fibromuscular dysplasia (FMD) is associated with 25%-86% of SCAD, underscoring the need for comprehensive vascular assessment in affected patients [3,4]. Additionally, hormonal fluctuations, particularly those related to pregnancy and menopause, have been implicated in the development of SCAD, as estrogen withdrawal may contribute to vascular fragility [3].

Early recognition of SCAD is critical to prevent misdiagnosis and inappropriate interventions. Coronary angiography remains the gold standard for diagnosis, allowing differentiation from other causes of ACS [5]. Conservative management is generally preferred, as percutaneous coronary intervention (PCI) in SCAD cases can exacerbate arterial dissection [5,6]. This case report highlights an unusual presentation of SCAD triggered by sexual activity. It discusses the importance of a tailored, conservative management approach to outcomes and addresses the role of adjunctive therapies, such as wearable cardioverter-defibrillators (WCDs), in mitigating the risk of adverse events during recovery.

## Case presentation

This study describes a 49-year-old woman with a history of essential hypertension, Class III obesity (BMI 41), and prediabetes. With respect to hormonal factors, the patient was multiparous and reported no history of hormonal contraceptive use or hormone replacement therapy. The exact menstrual cycle phase at presentation was not clearly documented, which is a limitation given the known association between hormonal variability and SCAD. She presented with acute retrosternal and left-sided precordial chest pain radiating to her neck and upper back, but not to her arm. The symptoms began shortly after sexual intercourse and were accompanied by dyspnea, nausea, and diaphoresis. Emergency Medical Services (EMS) were contacted, and she was transported to the emergency department (ED). An EKG performed by EMS showed ST-segment elevation, prompting activation of the cardiac catheterization lab en route. During transportation, the patient was immediately given an aspirin loading dose and two sublingual nitroglycerin doses. Upon arrival, her symptoms had improved.

**Figure 1:**
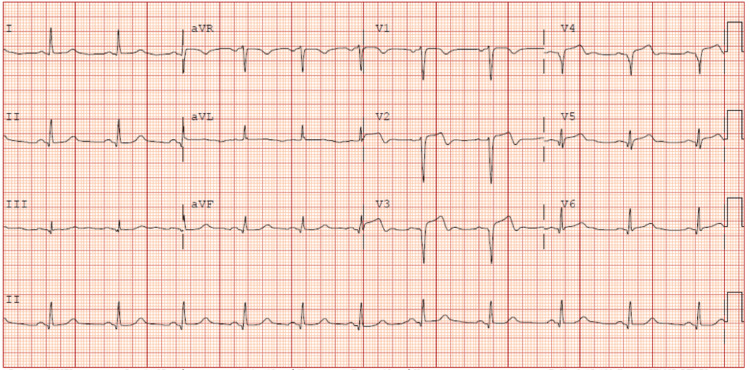
Initial EKG revealing ST-segment elevations in V2-V4 aVR: augmented vector right; aVL: augmented vector left; aVF: augmented vector foot

Her vital signs were stable, with a blood pressure of 123/73 mmHg, a heart rate of 68 beats/minute, a respiratory rate of 12 breaths/minute, and an oxygen saturation of 98% on room air. However, her rhythm alternated between sinus rhythm and accelerated idioventricular rhythm. ECG in the ED revealed ST-segment elevations in leads V2-V4 without reciprocal changes, as seen in Figure 1. The patient was immediately started on a heparin drip, nitroglycerin, aspirin, and atorvastatin. The differential diagnoses included STEMI, Takotsubo cardiomyopathy, vasospastic angina, pulmonary embolism, and SCAD.

Bedside echocardiography demonstrated a mildly reduced left ventricular ejection fraction (LVEF) of approximately 40%, with apical and distal septal hypokinesis and no pericardial effusion. A formal echocardiogram showed an LVEF of 35%-40%, moderate global hypokinesis, and regional hypokinesis of the anteroseptal and apical walls, as shown in Videos 1, 2. Laboratory findings included leukocytosis with a white blood cell count of 16.7 k/cmm (normal: 4.50-11.00 k/cmm) and a troponin level of <0.01 ng/mL. The observed leukocytosis is most likely attributable to a physiological stress response associated with acute myocardial injury rather than an underlying infectious process. The patient did not exhibit refractory chest pain after administration of nitroglycerin.

**Video 1:** TTE showing an LVEF of 35%-40% with moderate global hypokinesis and regional hypokinesis of the anteroseptal and apical wall. The Impella device was located in the left ventricle. The distance from the aortic valve annulus to the inlet is 2.20 cm. The RV cavity was within normal size. No evidence of pericardial effusion was seen TTE: transthoracic echocardiogram; LVEF: left ventricular ejection fraction; RV: right ventricle

**Video 2:** TTE with a definitive two-chamber view, showing an LVEF of 35%-40% with moderate global hypokinesis and regional hypokinesis of the anteroseptal and apical wall. No evidence of pericardial effusion was seen TTE: transthoracic echocardiogram; LVEF: left ventricular ejection fraction

**Figure 2:**
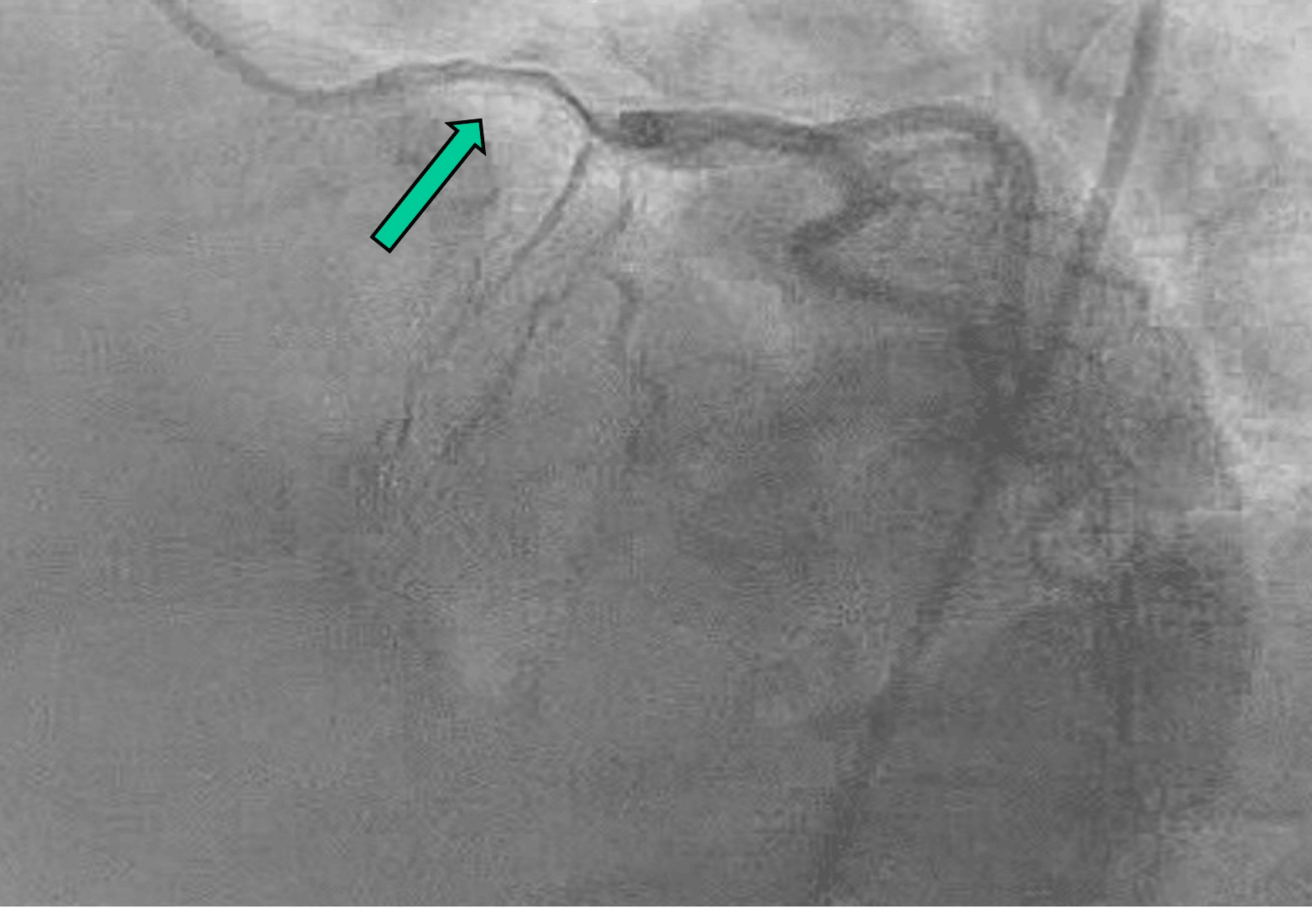
Coronary angiography showing diffuse narrowing of the LAD from the origin to the mid vessel, consistent with SCAD LAD: left anterior descending; SCAD: spontaneous coronary artery dissection

Cardiac catheterization revealed SCAD involving the entire course of the left anterior descending (LAD) artery, extending retrograde into the left main (LM) trunk and proximal left circumflex (LCx), with subintimal hematoma seen in Figure 2. Given the presence of subintimal hemorrhage and the associated risk of procedural complications, PCI was deemed clinically inappropriate. After evaluation, the decision was made to proceed with emergent coronary artery bypass grafting (CABG) due to the threatened appearance of her LM trunk. Given the initial concern for LM involvement, emergent CABG was considered. However, due to concurrent operative demands, immediate surgical availability was delayed. During this interval, an Impella device (Abiomed, Danvers, MA) was placed to provide temporary mechanical circulatory support and mitigate the risk of hemodynamic compromise. Following transfer to a tertiary care center, the patient remained hemodynamically stable without ongoing ischemia. After multidisciplinary reassessment, the decision to defer CABG was made based on clinical stability and the known risks of surgical and percutaneous intervention in SCAD, rather than logistical factors alone.

Following further evaluation, the Impella device was removed, and her condition improved to the extent that surgery was no longer necessary. She was managed conservatively without further interventions. During her hospitalization, she remained stable and was discharged on optimized medical therapy. At discharge, she was prescribed an increased dose of losartan, metoprolol succinate, spironolactone, and dual-antiplatelet therapy with aspirin and clopidogrel.

Additionally, she was provided with a WCD (LifeVest, ZOLL Medical Corporation, Chelmsford, MA) due to her reduced LVEF and elevated risk of ventricular arrhythmias. The LifeVest was intended as a temporary measure while awaiting outpatient echocardiography to reassess her ejection fraction and determine the need for a permanently implantable cardioverter-defibrillator (ICD).

Telephone follow-up confirmed progressive improvement in physical activity tolerance and resolution of chest pain. Follow-up echocardiography was performed within one month after discharge and demonstrated improvement in LVEF to 40%-45%, with mild residual left ventricular systolic dysfunction and persistent hypokinesis of the distal apical, anteroseptal, and inferoseptal walls. The patient continues to follow up with cardiology for ongoing management and reassessment of ICD candidacy.

## Discussion

SCAD is estimated to account for approximately 0.2%-4% of all ACS cases [1]. It predominantly affects women between the ages of 45 and 55 and is responsible for nearly 30% of ACS presentations in women under 50 [2]. Unlike atherosclerotic ACS, SCAD is a nonatherosclerotic process characterized by separation of the coronary arterial wall, leading to intramural hematoma formation or intimal disruption, ultimately compromising coronary blood flow and resulting in myocardial ischemia [1].

In the present case, a 49-year-old woman developed SCAD immediately following sexual activity, a recognized but relatively uncommon physical trigger. Her presentation with acute chest pain, ST-segment elevations, and reduced LVEF is consistent with previously described SCAD presentations, though the temporal association with intercourse highlights the role of acute hemodynamic and shear stress in precipitating arterial wall disruption. Sexual activity has been increasingly recognized as a precipitating stressor due to transient increases in heart rate, blood pressure, and vascular wall stress, all of which may contribute to dissection in susceptible individuals [7,8].

SCAD has been classified into three angiographic subtypes. Type 1 SCAD demonstrates a classic double-lumen or contrast-staining pattern, while Type 2, the most common subtype, presents as diffuse, smooth arterial narrowing that typically extends ≥20 mm [3]. Type 3 mimics atherosclerosis and often requires intracoronary imaging for diagnosis [3]. Our patient demonstrated a Type 2 SCAD pattern, with diffuse involvement of the LAD artery extending retrogradely into the LM and LCx arteries. This extensive, multivessel involvement is less common and represents a higher risk phenotype, particularly given LM involvement, which has been reported in approximately 12% of SCAD cases [4,6]. Additionally, retrograde propagation, as observed in our patient, occurs in fewer than 10% of cases and is associated with a more extensive intramural hematoma burden [5-7].

SCAD is strongly associated with both intrinsic vascular abnormalities and external stressors. FMD is present in 25%-86% of patients, emphasizing the importance of screening for systemic arteriopathies [8]. Hormonal influences also play a critical role, particularly in women during periods of hormonal fluctuation such as pregnancy, perimenopause, or the luteal phase of the menstrual cycle [9-11]. In this case, the patient had no known history of FMD or exogenous hormone use but was multiparous and experienced the event shortly before menstruation, suggesting a potential contribution of hormonal variability to vascular fragility. Furthermore, unlike many SCAD patients who lack traditional cardiovascular risk factors, this patient had comorbid hypertension, class III obesity, and prediabetes, which may have contributed to additional vascular stress and susceptibility.

Management of SCAD differs significantly from that of atherosclerotic ACS and is primarily conservative [12,13]. In our patient, initial treatment followed standard ACS protocols, including dual antiplatelet therapy, anticoagulation, and beta-blockade. However, coronary angiography revealed extensive dissection with subintimal hematoma, prompting avoidance of PCI due to the risk of propagation. Notably, although emergent CABG was initially considered, given LM involvement, the patient remained hemodynamically stable while receiving temporary mechanical circulatory support with an Impella device. This stabilization allowed for reassessment, and ultimately, conservative management was successfully pursued without surgical intervention. This clinical course highlights the dynamic nature of SCAD management and reinforces that even high-risk anatomical presentations may stabilize with supportive care.

The use of a WCD in this case was guided by the patient’s reduced LVEF (35%-40%) and the associated risk of malignant ventricular arrhythmias. WCDs provide temporary protection during myocardial recovery and have demonstrated benefit in high-risk populations [14,15]. In this patient, the WCD served as a bridge while awaiting reassessment of ventricular function and potential need for ICD placement. Follow-up demonstrated clinical improvement, supporting the role of conservative therapy combined with close monitoring.

This case underscores several important clinical considerations. First, SCAD should be considered in women presenting with ACS, particularly when triggered by physical or emotional stressors such as sexual activity. Second, angiographic recognition of SCAD subtypes is critical to avoid inappropriate interventions that may worsen outcomes. Third, even in cases of extensive multivessel involvement with LM extension, conservative management may be appropriate when the patient is hemodynamically stable. Finally, adjunctive therapies such as mechanical circulatory support and WCD use can play a crucial role in bridging patients through the acute phase of illness.

Overall, this case contributes to the growing body of literature emphasizing individualized, patient-centered management in SCAD, integrating clinical presentation, angiographic findings, and hemodynamic status to optimize outcomes.

Limitations

This case report has several limitations. As a single case, the findings cannot be generalized to broader patient populations. Although early clinical improvement was documented, follow-up duration was relatively short, limiting assessment of long-term outcomes and recurrence risk. Additionally, comprehensive evaluation for underlying arteriopathies, including FMD, and genetic testing were not performed during hospitalization. While follow-up echocardiography demonstrated improvement in left ventricular function, longer term imaging data remain limited.

## Conclusions

SCAD remains an important but underdiagnosed cause of ACS, particularly in middle-aged women with minimal traditional cardiovascular risk factors. Our case highlights the need to recognize SCAD in atypical clinical scenarios to avoid misdiagnosis and unnecessary interventions. The management of SCAD should prioritize conservative treatment strategies, including beta-blockers and dual antiplatelet therapy, while avoiding PCI unless hemodynamically necessary. Additionally, WCDs prevent fatal arrhythmias during myocardial recovery in patients with significantly reduced LVEF. The interplay between hormonal influences, vascular arteriopathies, and physical stressors underscores the need for further research into SCAD pathophysiology and optimal long-term management strategies. Multidisciplinary collaboration remains essential in ensuring comprehensive care and favorable outcomes for SCAD patients.
